# The supplementation of female dogs with live yeast *Saccharomyces cerevisiae* var. *boulardii* CNCM I-1079 acts as gut stabilizer at whelping and modulates immunometabolic phenotype of the puppies

**DOI:** 10.3389/fnut.2024.1366256

**Published:** 2024-04-12

**Authors:** Quentin Garrigues, Amélie Mugnier, Sylvie Chastant, Flavie Sicard, Jean-Charles Martin, Ljubica Svilar, Mathieu Castex, Manuel Guillermo Ramis-Vidal, Nicoletta Rovere, Laurine Michaud, Pauline David, Elodie Mansalier, Ana Rodiles, Hanna Mila, Emmanuelle Apper

**Affiliations:** ^1^NeoCare, ENVT, Université de Toulouse, Toulouse, France; ^2^Lallemand SAS, Blagnac, France; ^3^Aix-Marseille Université, C2VN, INRAE, INSERM, Marseille, France; ^4^CriBioM, Aix Marseille Université, Marseille, France; ^5^Department of Animal Production, Faculty of Veterinary Medicine, University of Murcia, Murcia, Spain; ^6^Instituo Murciano de Investigación en Biomedicina (IMIB), Murcia, Spain; ^7^Department of HASFS, VESPA, University of Veterinary, Milan, Italy

**Keywords:** yeast, maternal programming, dog, puppy, immunometabolic phenotype, *Saccharomyces cerevisiae boulardii*

## Abstract

Time around parturition is a stressful period for both bitches and their puppies. The use of probiotics has been proposed, e.g., in pigs, to improve health status of sows, their reproductive performances and in turn, the health and performance of their progeny. The objective of the present study was to evaluate the impact, on both dams and puppies, of a supplementation of bitches with the live yeast *Saccharomyces cerevisiae* var. *boulardii* CNCM I-1079 (SB-1079) during the second part of the gestation and the lactation period. A total of 36 bitches of medium and large-sized breeds were enrolled. They were divided into two groups, one of which received 1.3 × 10^9^ colony forming units of live yeast per day. At dam’s level, SB-1079 yeast shaped a different microbiota structure between the two groups just after whelping, impacted alpha diversity and some plasma metabolites related to energy metabolism. Regarding reproductive performances, SB-1079 improved gross energy of the colostrum (1.4 vs. 1.2 kcal of ME/g) as well as the concentration of protein in milk at Day 7 after parturition (10.4 vs. 7.6%). SB-1079 also reduced the odds of having low birth weight in the litter. At puppy’s level, a modulation of immunometabolic phenotype is suggested by the observation of increased growth rates during the early pediatric period (i.e., between 21 and 56 days of life, 225 vs. 190%) and a decrease of the IL-8:IL-10 ratio after vaccination against rabies (4.2 vs. 16.9). Our findings suggest that SB-1079 supplementation during gestation and lactation has the potential to enhance health of bitches and in turn health of puppies through maternal programming.

## Graphical abstract

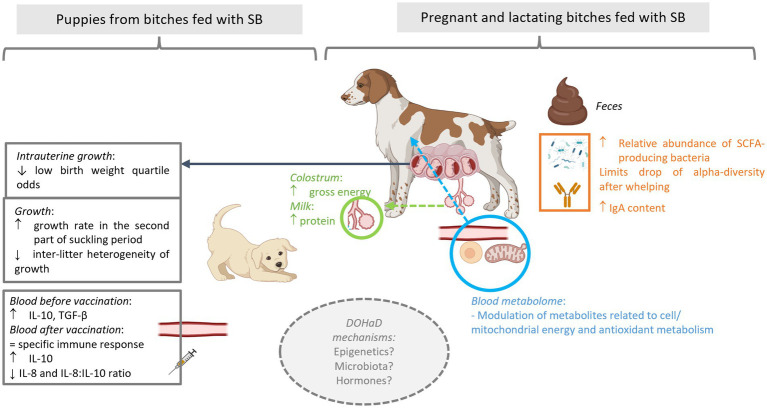

## 1 Introduction

Gestation, *peripartum* period and lactation are challenging times for dogs. Bitches must cope with rapid changes in their physiological state (1, 2) and puppies must adapt quickly from a protected intrauterine life to an extrauterine life. How the mother manages these periods is essential to ensure her good health but also to optimally support survival, health and well-being of the puppies (3–5). For example, adequate maternal behavior and sufficient colostrum/milk production are essential, not only for puppy survival but also for their growth until weaning. In addition, it was suggested that, as in humans, early events during the fetal and neonatal life of a dog may have long-term impacts on its health. In humans, this concept of the Developmental Origins of Health and Disease (DOHaD) was supported by a large body of epidemiological and experimental data since the first hypothesis in the 1990s (6). More recently some evidence regarding the long-term effects of early life exposures was also described in dogs (7) with, e.g., an increased risk of overweight among low birthweight (LBW) puppies (8).

Given the link between reproduction and nutrition, an appropriate nutritional strategy contributes to improve the health status of dams, their reproductive performances and in turn, the health of their litters at short and long-term (9–12). Probiotics, and particularly *Saccharomyces cerevisiae* var. *boulardii* (SB), have gained extensive attention in animal nutrition and have been demonstrated to modulate digestive microbiota and support the digestive processes (13, 14). More precisely, SB exerts a trophic effect on the intestinal microbiota, resulting in beneficial effects on the microbial community diversity, structure, and support of beneficial microbes and microbial functions. Mechanisms at play are likely to combine oxygen consumption, pathogen binding, production of short-chain fatty acids (SCFA, including acetic acid), production of polyamines, B-vitamins and production of specific proteases (15). This trophic effect on the microbiota (“gut microbiota stabilizer” effect) is expected to be responsible for most of the subsequent benefits measured in the intestine and at systemic level, even if a direct crosstalk between the yeast and the host’s cells cannot be ruled out. Those beneficial effects could, in turn, have a positive impact on welfare and performance of the animals. Recent scientific evidence on the use of SB demonstrated the involvement of maternal programming in sows and piglets through the dietary use of live microbial strains administrated during gestation and lactation (13, 16, 17). More precisely, feeding SB during gestation and lactation improves digestive health of the sows through an impact on intestinal transit and a stabilization of gut microbiota at farrowing, by promoting the relative abundance of fiber degrader bacteria while decreasing the presence of undesirable bacteria such as *Campylobacter*. Furthermore, SB is demonstrated as benefiting sows’ performances as well as immune status and performances of their piglets (16–18).

Like in swine, canine species are polytocous, and the female (bitch) can give birth to multiple puppies in the same litter. Another similarity with swine is that puppies are born hypogammaglobulinemic, with limited glucose reserves and hypothermic, making the mother and her nursing capacities pivotal for their survival (19). Moreover, just like in piglets, birth weight is critical for the newborn dog, as low birth weight puppies present considerably increased risk of death during neonatal period (20, 21). Despite the growing interest of the scientific community regarding dog’s microbiome, literature regarding the impact of live yeast supplementation on reproductive performance in canine species is scarce. The objective of this study was thus to evaluate the effect of live yeast *S. boulardii* CNCM I-1079 supplementation of the pregnant and lactating bitches on their microbiota, health, and reproductive performance and on growth and immune parameters of puppies. The hypothesis is that the “maternal programming” mediated effect observed in sows and their progeny could be extended to the canine species.

## 2 Materials and methods

### 2.1 Ethics statement

The animal study was reviewed and approved by the local ethical committee (Comité d’Éthique en Expérimentation Animale, Science et Santé Animale n°115; reference number: SSA_2020-004, Toulouse, France). The experimental protocol followed current applicable guidelines for the care and use of animals. Written informed consent was obtained from the owner of the kennel for the participation of its animals in this study.

### 2.2 Experimental design

#### 2.2.1 Animals and housing

This study was conducted in a single breeding facility. Thirty-six bitches from medium (> 15 and ≤ 25 kg) or large-sized (> 25 kg) breeds (Supplementary Table S5) were selected on day 28 of gestation (G28) after a positive pregnancy diagnosis performed by the veterinary practitioner with a portable ultrasound machine (G0 = day of ovulation determined by blood progesterone assay). They were randomly divided into two groups according to the breed size format, the age at mating, parity, body condition score and faecal score. From the time of inclusion, one group was supplemented with placebo capsules (Control, *n* = 18) and the other group with capsules containing the live yeast (Yeast, *n* = 18). Then, the follow-up of the dams and their puppies after whelping lasted until separation of the puppies, around 56 days *post-partum* (DPP56). All puppies included were born by natural delivery (i.e., no caesarean section) and remained with their mothers during the entire experiment allowing them to suckle freely.

Dogs were subjected to the same housing conditions with wood shaving as bedding material all the time. From ovulation to 8 days before whelping, bitches were kept in individual outdoor pens. Then, until 35 days *post-partum* (DPP35), they were housed in individual pens in the maternity building equipped with a floor heating system and heating lamps. Finally, dams and litters were moved to individual pens in the pre-weaning building of the breeding facility until the end of the trial. All animals were vaccinated and dewormed following the veterinary protocol in place at the breeding facility (Supplementary Table S1).

#### 2.2.2 Diets and probiotic supplementation

In order to fulfill the recommendations of the National Research Council (NRC) (22) linked to physiological stages of the bitches, two petfood manufactured by CRUSTY FOOD SAS (Montardit, Verteuil d’Agenais, France) were used. From G0 until G28, bitches received Diet 1, a complete extruded food containing wheat, peas, dehydrated poultry proteins, poultry fat, corn gluten, poultry protein hydrolysate, rice bran, meat hydrolysate, dehydrated lamb proteins, beet pulp, minerals and fish oil as feed materials; and trace minerals, vitamin A, vitamin D3 and vitamin E as nutritional feed additives. Then, from G28 to the end of the experiment (DPP56), Diet 2 was fed. It contained dehydrated poultry proteins, rice, wheat, corn, animal fat, rice bran, meat hydrolysate, corn gluten, beet pulp, minerals and fish oil as feed materials; and trace minerals, vitamin A, vitamin D3 and vitamin E as nutritional feed additives. Nutritional values of both diets are available in Table 1. Diet 2 was made available to puppies once they reached 3 weeks of age, after a transition of around one week with the same diet mixed with water. During the experiment, all dogs had *ad libitum* access to water.

**Table 1:** Analysis of nutritional values of the feeds used in the study.

Parameter	Feed	
	Diet 1	Diet 2
Moisture, %	5.7	6.3
Crude ash, % as fed	8.7	9.3
Crude protein, % as fed	30.8	28.4
Ether extract^a^, % as fed	15.1	16.8
Crude fibre, % as fed	2.7	<2
Carbohydrates^b^, %	37.0	37.7
Vitamin E, mg/kg as fed	124	114
Vitamin A, IU/kg as fed	7,670	7,330
Vitamin D, IU/kg as fed	1,080	1,000
Metabolizable energy^c^, kcal/100 g	385.8	408.6

a Crude fat.

b Carbohydrate was calculated from equation: %Dry Matter−(%Ether extract + %Crude Protein + %Crude Ash + %Crude fibre).

c Metabolizable energy was calculated from equation (22, 24).

The additive tested was *Saccharomyces cerevisiae* var. *boulardii* CNCM I-1079 (Levucell SB^®^, Lallemand SAS, Blagnac, France; SB-1079) given in 400-mg vegetal capsules (hydroxypropylmethylcellullose capsule size 1 clear, Suheung Co, South Korea) containing 6.25% yeast (measured at 6.4 × 10^8^ colony-forming unit (CFU) of yeast/capsule), 92.75% potato starch and 1% silicic acid. Each dam in the Yeast group received two capsules per day (i.e., 1.3 × 10^9^ CFU of yeast/day), one in the morning and one in the evening, in a bullet of dry food softened in hot water then turned into a bullet, made from the diet at the time (Diet 2 from G28) to ensure full consumption. The Control group was fed the same capsules in a bullet of wet petfood but without the yeast.

### 2.3 Sampling and measurements

**Figure 1:**
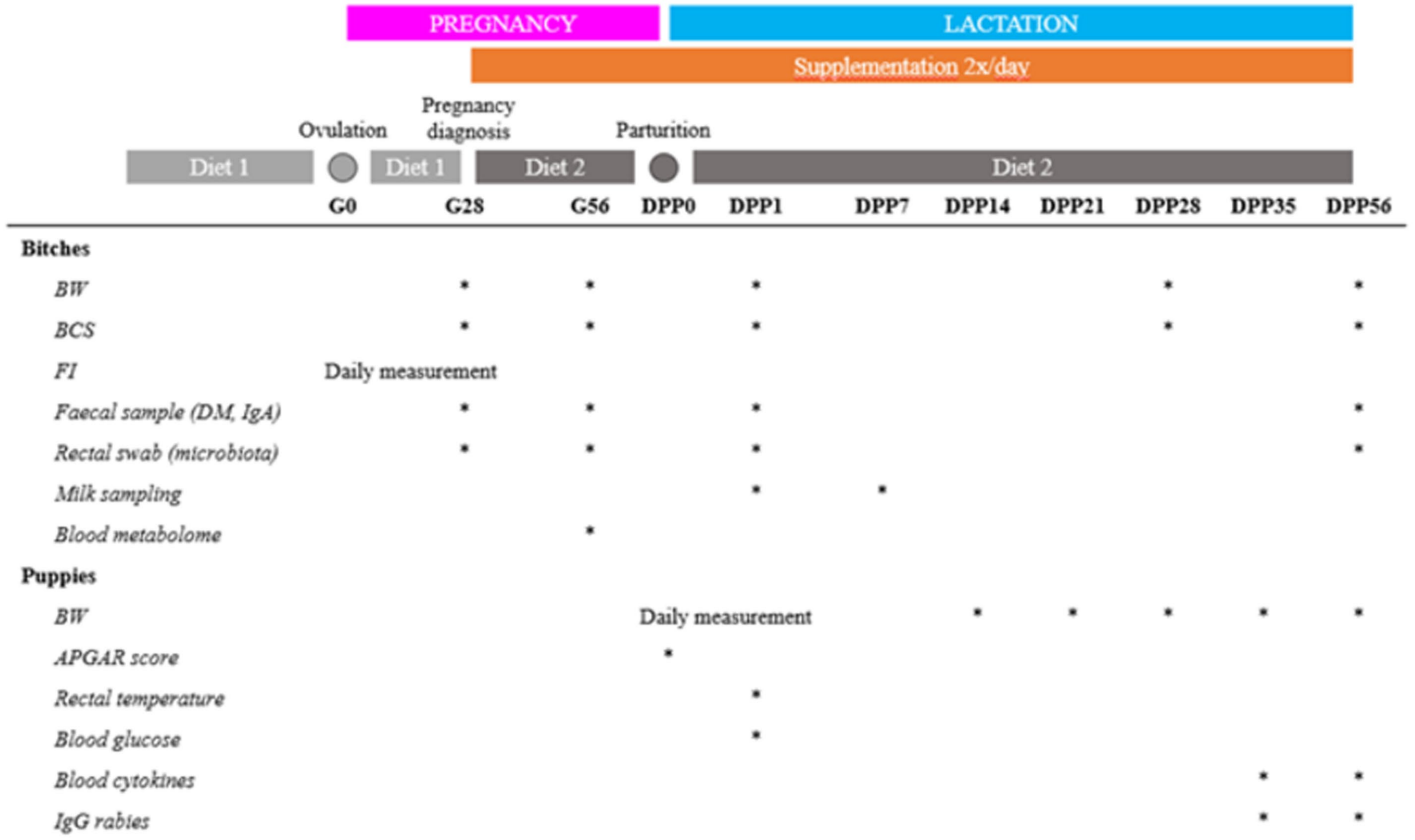
Schematic of the trial design. BW = body weight (kg), BCS = body condition score, FI = food intake, DM = dry matter, Gx = day x of the gestation, DPPy = day y post-partum.

To evaluate the impact of live yeast supplementation, various parameters were measured on the dams and their puppies (Figure 1).

#### 2.3.1 Parameters evaluated at dam level

##### 2.3.1.1 Zootechnical performances of dams

Individual body weight (BW) and Body Condition Score [BCS, 9-points scale (23)] of dams were recorded at G28, G56, DPP1, DPP28 and DPP56. The quantity of kibbles distributed before and remaining after a meal was recorded daily from G28 to the end of the experiment. Individual food intake (FI) was obtained by calculating the difference between the quantity of kibbles distributed daily and the quantity of kibbles remaining at the end of each day.

##### 2.3.1.2 Gut health related parameters

Feces were collected for three consecutive days at four time points: G28, G56, DPP1 and DPP56, pooled for each animal and stored at −20°C before further analysis to evaluate faecal dry matter (DM) and immunoglobulin A concentration (IgA). In addition, at the same time points, a faecal swab was collected and stored at −80°C to perform 16S rRNA amplicon sequencing and determine faecal microbiota composition.

##### 2.3.1.3 Blood metabolome

Blood was collected from the cephalic vein before whelping at G56 on a heparinized tube. The blood was centrifugated (16,000 RCF for 10 min) to isolate the plasma, and two aliquots of 250 μL each were put in the Eppendorf tubes and stored at −80°C for further analysis.

##### 2.3.1.4 Reproductive performances

For each dam, reproductive performances were evaluated through litter composition (number of born alive and stillborn puppies), losses (number of puppies dying from birth to DPP56) and colostrum/milk quality. Colostrum and milk were collected at DPP1 and DPP7, respectively, to determine nutritional content, IgG (for colostrum) and IgA concentrations. Before collecting the milk, an intramuscular administration of 1 or 2 UI of ocytocin was performed.

#### 2.3.2 Parameters evaluated at puppy level

Each of the 254 puppies from the 36 litters born alive were identified at birth using a colored collar.

##### 2.3.2.1 Newborn viability

APGAR score was recorded immediately after delivery except for puppies born between 9 p.m. and 4 a.m. A score of 7–10 means no distress, 4–6 means moderate distress and 0–3 means severe distress (25). Bodyweight at birth (BWb) was measured in the first 12 h after birth using a digital scale (EB3 Series, Ohaus, Parsippany, NJ, United States, maximum capacity of 5 kg, precision ±0.1 g). To deal with the variability of BWb depending on the breed (20, 26), puppies were categorized into quartile groups based on their breed and BWb (20, 21). Quartile 1 (Q1) included the 25% puppies with the lightest BWb, while quartile 4 (Q4) included the 25% heaviest. Threshold values for the targeted breeds (Supplementary Table S2) were defined based on the 4,010 BWb registered in the breeding facility during the last seven years. At DPP1, rectal temperature and blood concentration of glucose were measured.

##### 2.3.2.2 Growth of puppies

In addition to weights as such, two growth rates (GR) were calculated [GR 0–21 and GR 21–56, Eq. (1)].


(1) $$GR\;x-y\;\left(\text{in}\%\right)=\left[\left(\begin{array}{l} \text{weight}\;\text{at}\;\\ \text{Day}\;y-\\ \text{weight}\;\\ \text{at}\;\text{Day}\;x \end{array}\right)\right]/\text{weight}\;\text{at}\;\text{Day}\;x\times 100$$

##### 2.3.2.3 Vaccination, serum specific IgG and cytokines

57 puppies selected according to their health status and BWb were included for cytokines and immune response analyses. At DPP35, puppies were vaccinated against rabies (Rabigen mono, Virbac). Just before vaccination (DPP35) and at the end of the trial (DPP56) blood samples were collected from jugular vein in dry tubes. The blood was centrifugated (16,000 RCF for 10 min) to isolate the serum, and two aliquots of 500-750 μL each were put in the microcentrifuge tubes and stored at −80°C for further analysis.

### 2.4 Chemical analyses and DNA extraction

#### 2.4.1 Chemical analyses of diets

The experimental diets were subjected to Weende (proximate) analysis. They were dried to a constant weight at 103°C to determine dry matter (DM, ISO 1442, 1997). Crude ash was determined by combustion at 550°C (ISO 936, 1998). Crude protein was calculated from Kjeldahl nitrogen (6.25 × N, ISO 5983-1, 2005). Crude fibre was analysed by acid-alkali digestion (ISO 5498, 1981), and crude fat was analysed using acid-hydrolysis followed by Soxhlet extraction (ISO 1443, 1973). Analysis was performed by an external lab (Upscience, Vannes, France).

#### 2.4.2 Faecal dry matter and IgA

200 g samples of pooled faeces were used to determine faecal DM using freeze-dried method (University of Bologna, Italy, internal protocol). IgA content in lyophilized faecal samples was carried out by using a commercial kit (dog IgA ELISA Quantitation Set, Bethyl Laboratories Inc., Montgomery, TX, United States; assay range: 15.6–1,000 ng/mL), following the procedure described by Zannoni et al. (27).

#### 2.4.3 Faecal microbiota

DNA was extracted from rectal swabs using Quick-DNA Faecal/Soil Microbe Miniprep Kit (Zymo Research, Irvine, CA, United States) following the manufacturer’s instructions. Quantification of extracted DNA was checked using a fluorometric method with Quant-iT^™^ PicoGreen^®^ dsDNA assay kit (Life Technologies, Carlsbad, CA, United States) measured via QuantStudio^™^3 Real Time PCR System (Thermo Fisher Scientific Inc., Waltham, MA, United States). The V3-V4 region of the 16S rRNA gene was amplified by PCR using universal primers 341F (CCTACGGGAGGCAGCAG) and 806R (GGACTACNVGGGTWTCTAAT). PCR amplicons were purified with HighPrep PCR system (MAGBIO GENOMICS, Gaithersburg, MD, United States) and used for library construction with the Illumina NEXTflex PCR-Free DNA sequencing kit (Bioo Scientific corp., Austin, TX, United States). Amplicon libraries were sequenced on an Illumina MiSeq 2,500 platform (Illumina, San Diego, CA, United States) at GeT-PlaGe INRAE Platform (Toulouse, France) for paired-end fragment sizes of 250 bp. All reagents used were molecular grade.

#### 2.4.4 Milk and colostrum composition

Protein, sugar and lipid assays from milk and colostrum samples were carried out in collaboration with the Department of Nutrition at the Smithsonian Institute in Washington using standard colostrum and milk analysis methods developed by Hood et al. (28). For the determination of dry matter, the colostrum and milk samples were weighed, dried in a forced convection oven for 3.5 to 4 h at 100°C and then reweighed. Total nitrogen was determined using the Kjeldahl method with a Dumas nitrogen gas analyser. The total amount of nitrogen was then multiplied by 6.38 to determine total protein (29). Sugars were determined by phenol-sulphuric acid colorimetric procedure using a standard range of lactose monohydrate (30) and read at 490 nm on a microplate reader (Model ELX808; BioTek, Winooski, VT). Fat content was determined using the Röse-Gottlieb method (28), which involves three sequential extractions with diethyl ether and petroleum ether after disaggregation of milk fat globules with ammonium hydroxide and ethyl alcohol. All above nutrient contents were expressed as the percentage of nutrient content per 100 g of colostrum or milk. The gross energy (GE) content of colostrum and milk were calculated from the values of protein, sugar and lipid content using Eq. (2) with fat, protein and sugar expressed as percentage (31).


(2) GE=9.11×fat+5.86×protein+3.95×sugar

This equation is likely to slightly overestimate GE because it does not correct for non-protein nitrogen. However, it has been verified against GE values measured by calorimetry (32). Finally, milk was stored at −20°C until IgA and IgG assays using a previously described and validated ELISA method (Dog IgG and Dog IgA ELISA Kits, Bethyl Laboratories, Montgomery, United States) (33, 34).

#### 2.4.5 Serum cytokines determination

The IL-8, IL-10, IFN-α, IFN-γ TNF-α and TGF-β concentrations in serum were tested by ELISA. The range of minimum detection, sensitivity and type of sample that could be used for each cytokine are listed in Supplementary Table S3. Once performed according to manufacturer instructions, the results were read in a spectrophotometer (Biochrom Anthos 2010 Microplate Reader, Biochrom LTD, Cambridge, UK) at the wavelength indicated for the dye used (450 nm). The quantification was made by means of a standard curve. In all cases will be included positive and negative controls (blanks).

#### 2.4.6 Specific IgG against rabies

Quantification of anti-rabies antibodies was assessed using fluorescent antibody virus neutralization (FAVN) test, as recommended in the OIE Terrestrial Manual 2018 (35). Prior to the test, 80 μL of puppies’ serum were diluted with 160 μL of Dulbecco’s Modified Eagle Medium (DMEM) with 10% of fetal calf serum. All puppies with levels of antibody equal to or above 0.5 UI/mL were considered protected.

#### 2.4.7 Metabolome

Polar and semi-polar metabolites were extracted from the plasma of the bitches by protein precipitation using methanol. 50 μL of plasma sample was homogenized in 200 μL of −20°C cold methanol. After shaking and one-hour incubation at −20°C, samples were vortex homogenized and centrifuged for 15 min at 4°C and 16,000 relative centrifugal force (RCF). Supernatants were than filtered through a 10 kDa centrifugal filter (VWR^®^, Rosny-sous-Bois, France, 10 kDa), dried under the gentle nitrogen stream and resuspended in 125 μL of water/acetonitrile/formic acid (90/10/0.1, v/v/v). Samples were transferred in to the 0.45 μm centrifugal filters (VWR^®^, 0.45 μm) and filtered for 15 min at 16000 RCF and 4°C. 50 μL of sample was transferred into the vials and stored at −80°C before the analysis. Quality control samples, pool samples were prepared by assembling an equal volume of each analyzed plasma sample. Blank samples were prepared following the same extraction protocol but for water instead of plasma.

All samples were analyzed using high performance liquid chromatography (Dionex UltiMate 3,000, Thermo Scientific, Bremen, Germany) hyphenated to a high-resolution mass spectrometry (Q-Exactive Plus hybrid mass spectrometer, Thermo Scientific). Samples were analyzed randomly, and pool samples were injected every five biological samples. Reverse and normal phases were used for the chromatographic separation. For reverse phase, a Hypersil Gold C18 (100 mm × 2.1 mm × 1.9 μm) (Thermo Scientific) column was used. Oven temperature was set at 40°C. The flow rate was maintained at 0.4 mL/min, and 0.1% formic acid solutions in water and acetonitrile were used in mobile phases A and B, respectively. A first minute at 0% of B in the isocratic elution was followed by ten minutes on a linear gradient to 100% B, which was then maintained in isocratic mode for two minutes. Gaining initial conditions in one minute was followed by two minutes column equilibration. For normal phase separation, a HILIC (Se-Quant, ZIC-HILIC Peek Coated 150 × 2.1 mm × 5 um, Merck) column was used. The oven temperature was kept at 25°C, and the flow rate at 0.25 mL/min. 16 mM ammonium formate in water was a mobile phase A, and 0.1% formic acid was mobile phase B. Chromatographic separation was done as follows. For first two minutes phase B was kept at 97%, then for 8 min decreased to 70%, for 5 min decreased to 10% and stayed isocratic for 2 min. After this gradient, the initial conditions were achieved in one minute and the column equilibration was achieved in nine minutes. Data acquisitions were obtained in a switching ion polarities mode in the *m/z* 80–100 range and with resolving power 35,000 FWHM (for *m/z* 200). Electrospray needle was kept at ±3.5 kV, S-lens RF level of 55 and a capillary temperature of 320°C. Sheath gas, auxiliary gas and sweep gas flow rates were maintained at 30, 8, and 0 arbitrary units, respectively. MS/MS spectra were acquired on a pool sample using High Collision energy Dissociation (HCD) and Data Dependent Analysis (DDA) method to obtain structural information for the large palette of metabolites.

Raw data were converted to mzXML files and processed using XCMS library in the R environment. Parameters applied for the data processing in each analysis mode are described in Supplementary Table S4.

Tables of each analyzing mode, containing the *m/z*, retention times and intensities of the signals detected in all analyzed samples, were than filtered (coefficient of variation <30% in the repeated QC samples) and normalized (Van-der-Kloet algorithm), and only stable peaks were submitted to the annotation using our in-house database containing the possible adducts *m/z* and retention times of around 1,300 endogenous metabolites. Attributed annotations were verified by comparing the reference and pool MS/MS spectra.

### 2.5 Statistical analyses

Statistical analyses were performed using R software (version 4.1.0) (36) for all parameters except microbiota for which QIIME2 (version 2020.2) (37) was used.

#### 2.5.1 Models used

The quality of the randomization based on the chosen criteria (age, BCS, faecal score and parity) was assessed using a nonparametric Wilcoxon-Mann–Whitney (WMW) rank test between Yeast and Control groups.

##### 2.5.1.1 Food intake, body weight, body condition score, faecal DM

The effect of the supplementation with SB-1079 on FI (total, during gestation and during lactation) was analysed using linear model with treatment, breed size, litter size and age of the dam as fixed effects (38). BW and BCS were also analyzed using linear mixed models with the same fixed effects plus time, interaction “time × treatment” and “dam” as random effect using lme4 and lmerTest packages (39, 40).

##### 2.5.1.2 Colostrum and milk composition

A linear model was used with treatment, breed size, litter size and dam’s age as fixed effects.

##### 2.5.1.3 Litter size and health related endpoints

Count data (i.e., number of puppies born, stillborn, number of weaned puppies) were analysed with generalized linear model (GLM) with a Poisson distribution. Treatment, breed size, litter size (except for number of puppies born) and age of the dam were used as fixed effects.

##### 2.5.1.4 Quartiles of birthweight

First, a Chi-Square analysis was performed to evaluate the repartition of puppies within quartiles between the treatment groups. Afterwards, a multinomial regression was performed using the treatment, the litter size, the age of the dam as fixed factors and individual/female dog as random effect with the ordinal package (note: breed size was not included in the models as this effect was considered in the variable construction).

##### 2.5.1.5 Growth parameters

The effect of the supplementation with SB-1079 on growth rate was analysed using a linear mixed model with treatment, breed size, litter size, age of the dam, quartile of puppies’ BWb as fixed effects, dam as random effect. This model allowed to handle correlation among puppies of the same litter and thus to avoid the pseudo-replication that using dogs of the same litter would cause (20, 41).

To adjust multivariate models, litter size was classified into three classes depending on the number of puppies born: less than 7 (Class 1), 7 or 8 (Class 2) and more than 8 (Class 3). Age of bitch at mating is an important factor associated to the reproductive performance (42, 43). It was included using three classes: ≤ 3 years (Class 1),[3; 4 years] (Class 2) and > 4 years (Class 3).

##### 2.5.1.6 Faecal IgA in dams, serum cytokines and specific IgG in puppies

For all those parameters, the yeast effect was evaluated using nonparametric WMW rank tests.

##### 2.5.1.7 Faecal microbiota

All bioinformatics analysis were done according to Garrigues et al. (44) in QIIME2 (version 2020.2) with Greengenes (gg-13-8-99-nb-classifier) using sklearn classifier method according to Bokulich et al. (45). To compare paired differences in alpha-diversity (e.g., the microbial diversity within the faecal ecosystem – i.e. intra-individual diversity of the faecal microbiota, evaluating using the Shannon index), a longitudinal analysis was done using a non-parametric Prentice signed-rank test within treatment and nonparametric WMW rank test between treatments. PERMANOVA on the Aitchison distance matrix was used to assess the benefits of the yeast on the beta-diversity (i.e., the interindividual diversity of the faecal microbiota) in QIIME2 (version 2020.2). Finally, linear discriminant analysis effect size (LEfSe) (46) from Galaxy was used to identify differences in taxonomy data between the two groups just after whelping and the average composition during the whole study.

##### 2.5.1.8 Metabolomics

Collected, filtered and annotated data from C18 and HILIC chromatographic separation and from positive and negative ionization modes of 83 plasma samples were merged in one non-redundant table containing 228 putatively identified molecules. When a metabolite was detected in both ionization modes or in both chromatographic modes (C18 and HILIC), the one chosen corresponded to the least coefficient of variation in the QC sample and to the highest raw intensity measured. A partial least-squares discriminant analysis (PLS-DA) was used to observe and discriminate bitches fed or not with SB-1079.

#### 2.5.2 Models’ construction and validation

Fixed effects and covariates were chosen for their biological relevance. Collinearity was assessed graphically to build the statistical model while a score called the variance inflation factor [VIF, (47)] was calculated to validate the final model. This score measures how much the variance of a regression coefficient is inflated due to multicollinearity in the model. The smallest possible value of VIF is 1 (absence of multicollinearity). As a rule of thumb, a VIF value that exceeds 5 indicates a problematic amount of collinearity (48).

For linear models and quantitative outcomes, adjusted means and standard errors (SE) obtained in the Control and Yeast groups, respectively, were reported, as well as the *p*-values for fixed effects. When required, Tukey *post-hoc* tests were performed (49). Assumptions of normality and homoscedasticity of the residuals were verified with the visual observation of studentized residuals’ plots, and with statistical tests of Shapiro and Bartlett for normality and homoscedasticity of the residuals, respectively. If assumptions were not fulfilled, a non-parametric Wilcoxon rank sum test was performed.

For all generalized linear models using a Poisson distribution (number of puppies), the appropriateness of the model to the data was assessed by Pearson residuals test. The dispersion of the residuals was evaluated by the ratio between the residual deviance and the number of degrees of freedom and by a dispersion test (50), testing the null hypothesis of equidispersion in Poisson GLM against the alternative of overdispersion. If surdispersion was detected, a model using a quasipoisson law was performed. *p*-value, incidence ratio, and 95% confidence interval are reported for each regression.

For multinomial regression (51), the appropriateness of the model to the data was assessed by Pearson residuals test. To confirm the importance of the yeast treatment (and other fixed effects) in the model, a likelihood ratio test was performed using nested models, one with and the other without the treatment (or the targeted fixed effect), with a comparison between different models to check the significant of each model tested. Dispersion parameter was also checked by the ratio between the residual deviance and the number of degrees of freedom which should be close to 1. *p*-value of comparison between Control and Yeast groups, odds ratio, and 95% confidence interval are reported.

For all the statistical procedures, an effect was considered as statistically significant with *p* < 0.05, a biological trend was noticed for *p* < 0.10.

## 3 Results

### 3.1 Description of the population studied

The final bitches’ cohort was composed of 4 and 5 medium-size and 14 and 13 large-size breeds enrolled in the Control and Yeast group, respectively (Supplementary Table S5). No statistical difference was detected for all the parameters considered for the randomization, confirming that the animals were homogeneously balanced between the two groups based on the chosen criteria (Supplementary Table S6).

### 3.2 Impact of the supplementation at dam’s level

#### 3.2.1 Bodyweight, body condition and food intake

No difference was observed between experimental groups at any time for BW, BCS and FI of the dams (all *p* > 0.10, Table 2). As anticipated due to their physiological status, a time effect was observed for BW, which was higher at the end of gestation (G56), lower at DPP1 and intermediate at G28, DPP28 and DPP56 (Table 2). BCS was stable all along the study except just after parturition (DPP1), where it transiently decreased in both groups.

**Table 2:** Average body weight, body condition score, gestation and lactation durations and food intake of bitches supplemented or not with *Saccharomyces cerevisiae* var. *boulardii* CNCM I-1079.

Parameters	LS means ± SE		*p*-value (fixed effect)					
	Control	Yeast	Treatment	Time	Treatment × Time	Age	Breed size	Litter size
BW, kg	29.3 ± 1.12	26.9 ± 1.11	0.117	<0.001	0.758	0.346	<0.001	0.684
BCS	4.96 ± 0.31	4.78 ± 0.31	0.667	<0.001	0.647	0.422	0.274	0.703
Gestation duration, d	61.6 ± 0.24	61.9 ± 0.23	0.355	NR	NR	0.122	0.017	0.702
Lactation duration^a^, d	58.6 ± 0.61	57.8 ± 1.53	0.650	NR	NR			
FI gestation, g/d	524 ± 25.7	529 ± 25.3	0.784	NR	NR	0.068	0.005	0.626
FI lactation, g/d	936 ± 75.0	838 ± 73.8	0.409	NR	NR	0.124	0.173	0.893
FI overall, g/d	784 ± 50.3	729 ± 49.5	0.523	NR	NR	0.077	0.071	0.823

a Wilcoxon-Mann–Whitney rank test (conditions for a linear model not fulfilled). Raw means are given in this case.

LS means = adjusted least square means, SE = standard error, BW = bodyweight, BCS = body condition score, FI = food intake, NR = non relevant (time effect was not tested), d = day.

#### 3.2.2 Gut health of the dams

For faecal DM, the interaction between time and treatment tended to be significant (*p* < 0.1). Indeed, a reduction of faecal DM over the course of the experiment was detected in the Control group while it remained stable in the Yeast group (−2% vs. 0%).

Faecal IgA concentrations averaged between G56 and DPP56 tended to be higher in the Yeast group compared to the Control group (23.3 ± 3.17 vs. 14.5 ± 1.98 mg/g, *p* < 0.1) with a significant difference at DPP1 (11.8 ± 2.83 and 23.0 ± 4.16 mg/g DM for Control and Yeast group respectively, *p* < 0.05). No time effect was observed on faecal IgA concentration (data not shown).

**Figure 2:**
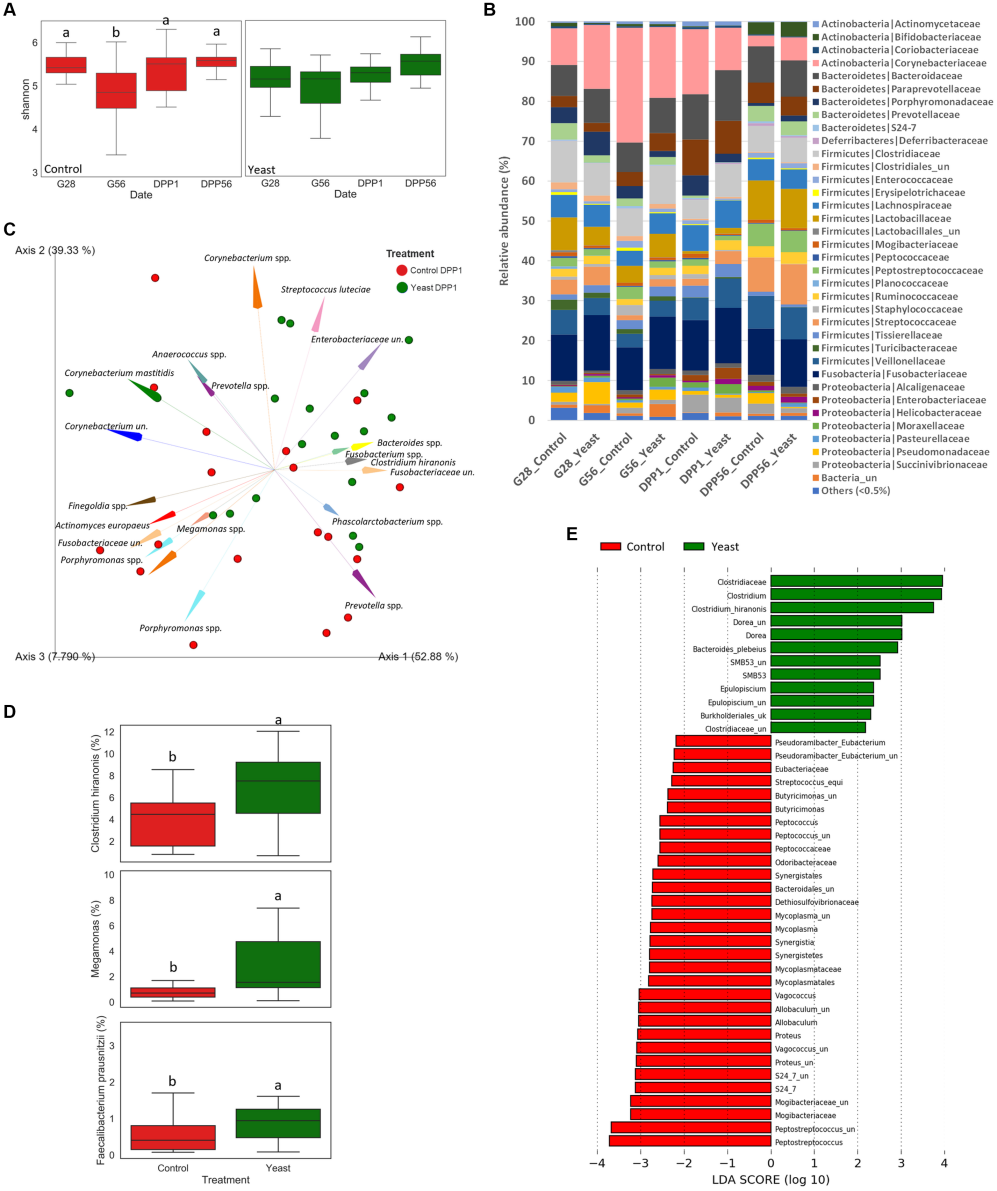
Impact of the supplementation with *Saccharomyces cerevisiae* var. *boulardii* CNCM I-1079 on gut health of bitches during the second part of the gestation and the lactation period. **(A)** Box and whiskers plots of the Shannon index evolution per treatment during the trial. Significant differences (in control group) are shown with different letters at the top of the boxplots (*p* < 0.05). **(B)** Phylum and family relative abundances (%) at the different time points for each group during the trial. **(C)** Aitchison distances characterizing the supplemented and the control groups at one day after parturition (DPP1) (*p* < 0.05) (un.: unknown genus or species). **(D)** Box plots of 3 key taxa relative abundances at one day after parturition for each group. Significant differences are shown with different letters et the top of the boxplots (*p* < 0.05). **(E)** Linear discriminant analysis effect size (LEfSe) for the overall period.

The evolution of the Shannon index as a marker of alpha diversity in the faecal microbiota differed between treatments across the experimental period (Figure 2A). In the Yeast group, the alpha diversity did not significantly change during the study period even if a gradual numerical increase was described during lactation. In particular, there was no marked reduction in the alpha diversity around whelping (G56) contrary to what was observed in the Control group. Indeed, significant differences were identified in the Control group between G28 and G56 and then G56 and DPP1.

Whatever the treatment and the time of sampling, the microbiota of the bitches was mostly composed of taxa belonging to the phylum Firmicutes, Bacteroidetes, Actinobacteria, Fusobacteria and Proteobacteria, and family Corynebacteriaceae, Fusobacteriaceae, Bacteroidaceae, Clostridiaceae, Veillonellaceae, Lactobacillaceae, Lachnospiraceae, Paraprevotellaceae and Streptococcaceae (Figure 2B). A PERMANOVA analysis showed that the beta-diversity significantly differed at DPP1 between Control and Yeast groups (Figure 2C; Table 3), where relative abundances of *Clostridium hiranonis*, *Faecalibacterium prausnitzii* and *Megamonas* were higher in the Yeast group compared with Control group (Figure 2D), together with *Dorea* and *Bacteroides plebeius* identified by LEfSe discriminant analysis (Supplementary Figure S1). These taxa were also detected across the entire study, suggesting a lasting effect of the yeast on the microbiota of dogs. It is also interesting to note that along the study, the microbiota of bitches in the Control group was characterized by higher relative abundances of *Proteus, Mycoplasma, Tissierellaceae*, Pasteurellaceae or *Vagococcus* compared to the Yeast group (Figure 2E).

**Table 3:** Effect of a supplementation with the additive on the beta-diversity using PERMANOVA on the Aitchison distance matrix at day 1 *post-partum* (DPP1)^a^.

Group comparison	Aitchison distance	Pseudo-*F*-value	*P*-value
**Aitchinson distance, DPP1**			
Control–control (within group)	1.945 ± 0.880	4.076	0.02
Yeast–yeast (within group)	1.634 ± 0.789		
Control–yeast (between groups)	1.984 ± 0.909*		
**Aitchinson distance, from G56 to DPP56**			
Control–control (within group)	1.953 ± 0.024	1.079121	0.338
Yeast–yeast (within group)	1.860 ± 0.016		
Control–yeast (between groups)	1.826 ± 0.013		

a *P* < 0.05 in the PERMANOVA test means that the distances between the two groups are significantly different.

#### 3.2.3 Plasma metabolome

In order to identify relevant metabolites allowing to discriminate, at G56, bitches fed or not with SB-1079, two successive PLS-DA were used. The first was used as a basis for fitting a more robust model by selecting the most discriminant metabolites, i.e., those with a variance importance in projection (VIP) score greater than 1.48. The final PLS-DA (R2Y = 0.562, Q2Y = 0.466) was adjusted with 1 latent variable and based on 23 metabolites. The result of cross-validated analysis of variance (CV-ANOVA; *p* < 0.01) indicated that the final PLS-DA model was valid and was able to discriminate between bitches of Control or Yeast group. It highlighted 23 discriminant metabolites (Supplementary Figure S2): xanthosine, N-acetylneuraminate, 2-aminopyridine-3-carboxylic acid, 2-hydroxybutyrate, L-alanine, 16:0-lysophosphatidylcholine, 18:0-lysophosphatidylcholine, indole-3-acetate, itaconate, 16:0-lysophosphatidylinositol, kynurenic acid, 4-methyl-L-glutamate, 2-hydroxyisocaproic acid, butyryl- and octanoyl-L carnitines, 3-hydroxybutanoate, 4-coumarate, 5-methylthioribose, O-acetylserine, ureidopropionate, creatine, creatinine and trans 4-hydroxy-L-proline.

#### 3.2.4 Reproductive performances

##### 3.2.4.1 Colostrum and milk composition

As shown in Table 4, gross energy of the colostrum was significantly higher in the Yeast group compared with the Control (*p* < 0.05) and a tendency of increased glucose concentration was noted (*p* < 0.1). In addition, the concentration of protein in milk was found significantly higher in Yeast than those in Control group (*p* < 0.05), while a trend of higher gross energy was also observed (*p* < 0.1).

**Table 4:** Impact of the supplementation with *Saccharomyces cerevisiae* var. *boulardii* CNCM I-1079 on composition of colostrum and milk (*n* = 18 per group).

	LS means ± SE		*P*-value			
	Control	Yeast	Treatment	Age	Breed size	Litter size
*Colostrum (DPP1)*						
**Dry matter, %**	21.0 ± 0.87	22.4 ± 0.89	0.375	0.669	0.022	0.909
**Crude protein, %**	8.96 ± 0.74	9.46 ± 0.76	0.396	0.912	0.507	0.013
**Sugars, %**	2.64 ± 0.19	3.08 ± 0.20	*0.059*	0.550	0.186	0.396
**Fat, %**	6.80 ± 0.61	7.78 ± 0.60	0.117	0.005	0.563	0.998
**Gross energy, kcal/g**	1.24 ± 0.60	1.39 ± 0.62	0.015	0.032	0.337	0.056
**IgA, g/L**	12.3 ± 3.05	16.3 ± 3.00	0.512	*0.058*	*0.063*	0.848
**IgG, g/L**	41.8 ± 7.39	44.0 ± 7.27	0.776	0.031	*0.062*	0.737
*Milk (DPP7)*						
**Dry matter, %**	20.8 ± 0.72	22.2 ± 0.65	0.306	0.456	0.043	0.551
**Crude protein, %**	7.64 ± 0.83	10.4 ± 0.76	0.042	0.003	0.316	0.592
**Sugars, %**	3.56 ± 0.02	3.46 ± 0.02	0.966	0.398	0.428	0.699
**Fat, %**	8.98 ± 0.71	9.53 ± 0.65	0.416	0.198	0.473	0.863
**Gross energy, kcal/g**	1.41 ± 0.09	1.61 ± 0.08	*0.093*	0.215	0.330	0.706
**IgA, g/L**	13.0 ± 2.60	18.1 ± 2.52	0.297	0.003	*0.083*	0.830

LS means = adjusted least square means, SE = standard error, DPP = day post-partum.

##### 3.2.4.2 Litter composition and puppy viability

**Figure 3:**
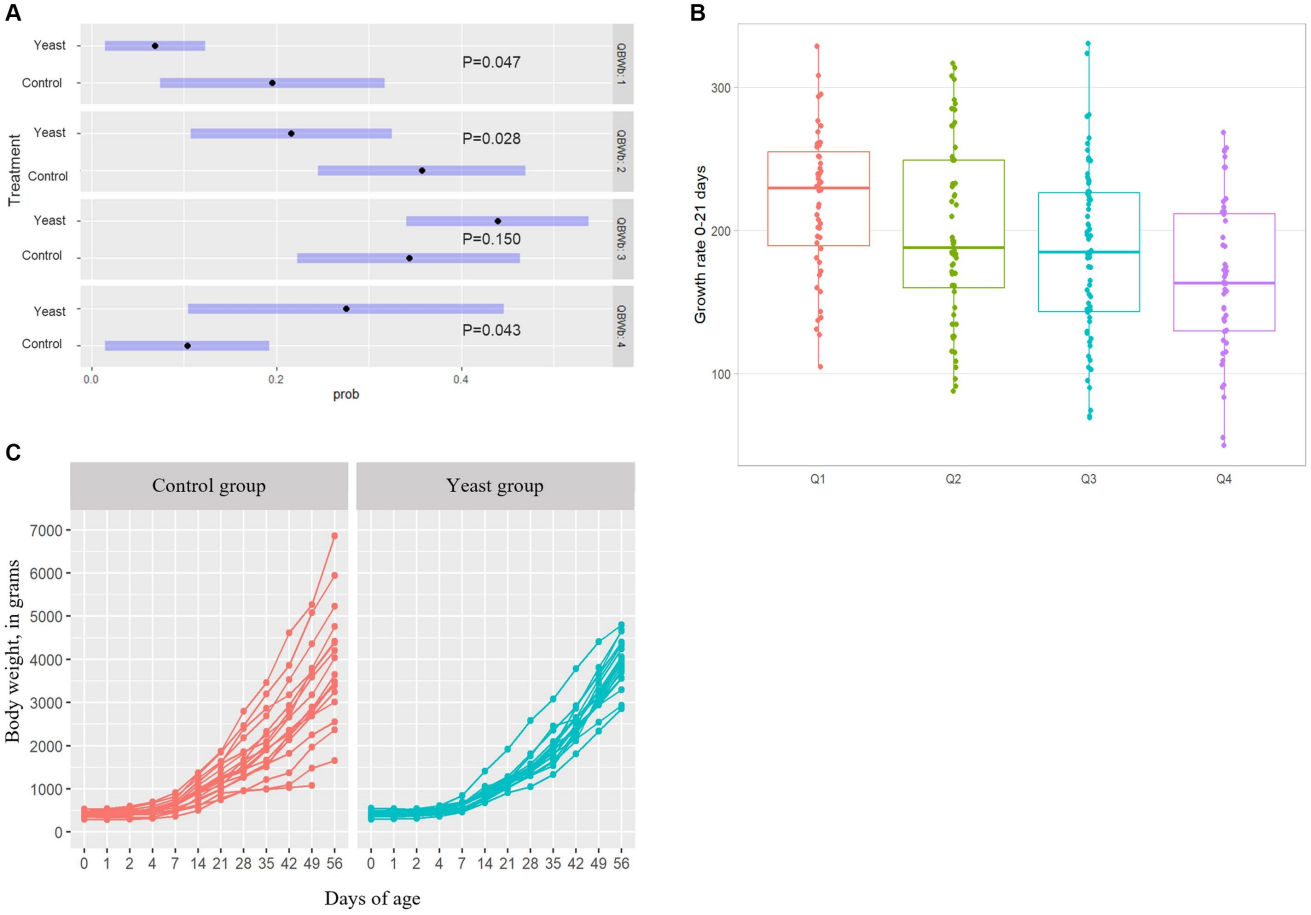
Impact of the supplementation of dams with *Saccharomyces cerevisiae* var. *boulardii* CNCM I-1079 on puppy’s growth. **(A)** Results of the multinomial regression showing that the odds of having puppies’ birth weight in other quartiles than Q1 and Q2 was higher in Yeast group compared with Control group. **(B)** Comparison of growth rates during the first three weeks of life depending on the birth weight quartile. **(C)** Evolution of average body weight per litter between birth and two months of age according to treatment groups.

In total 284 puppies were born during the study (142 in each group) with 254 puppies born alive. As shown in Table 5, no difference in the number of puppies born or in the number of stillborn puppies was evidenced between the two groups. However, the multinomial regression demonstrated that the odds of having puppies with BWb from Q1 and Q2 was lower in Yeast compared with Control group (Table 6; Figure 3A). A litter effect was also observed, with the largest litters having greater odds to have LBW puppies (Q1).

**Table 5:** Impact of the supplementation of the dam with *Saccharomyces cerevisiae* var. *boulardii* CNCM I-1079 on litter size, number of puppies born alive, and number of puppies weaned and mortality at weaning (*n* = 36 litters and 254 born alive puppies).

Item	Control	Yeast	IRR ± SE	95% CI	*P*-value
*Litter size*					
**Intercept**			7.86 ± 1.13	6.19–9.85	<0.001
**Treatment (control as reference)**	142	142	1.01 ± 1.13	0.80–1.28	0.935
**Age (class 1 as reference; ≤3 y)**					
Class 2 (>3 ≤ 4 years)	124	78	0.99 ± 1.16	0.74–1.32	0.966
Class 3 (>4 ≤ 6 years)	124	82	1.06 ± 1.15	0.80–1.40	0.690
**Breed size (large as reference)**	216	68	0.94 ± 1.15	0.71–1.23	0.656
*Stillborn puppies*					
**Intercept**			0.30 ± 2.17	0.05–1.17	0.125
**Treatment (control as reference)**	17	13	0.78 ± 1.47	0.36–1.65	0.509
**Age (class 1 as reference; ≤3 y)**					
Class 2 (>3 ≤ 4 years)	5	8	2.36 ± 1.78	0.78–7.86	0.136
Class 3 (>4 ≤ 6 years)	5	17	4.89 ± 1.67	1.92–14.9	0.002
**Breed size (large as reference)**	27	3	0.41 ± 1.92	0.09–1.28	0.174
**Litter size (class 1 as reference, <7 puppies born)**					
Class 2 (7–8 puppies born)	3	8	1.08 ± 2.03	0.29–5.16	0.918
Class 3 (> 8 puppies born)	3	19	1.87 ± 1.96	0.56–8.56	0.352
*Number of weaned puppies*					
**Intercept**			4.33 ± 1.258	2.71–6.66	<0.001
**Treatment (control as reference)**	111	110	0.97 ± 1.148	0.74–1.27	0.823
**Age (class 1 as reference; ≤3 y)**					
Class 2 (>3 ≤ 4 years)	108	61	0.95 ± 1.178	0.68–1.30	0.746
Class 3 (>4 ≤ 6 years)	108	53	0.79 ± 1.186	0.56–1.10	0.168
**Breed size (large as reference)**	161	61	1.22 ± 1.169	0.90–1.65	0.194
**Litter size (class 1 as reference, <7 puppies born)**					
Class 2 (7–8 puppies born)	30	82	1.50 ± 1.242	0.99–2.33	0.062
Class 3 (> 8 puppies born)	30	110	1.68 ± 1.232	1.13–2.57	0.013

IRR = Incidence Rate Ratio, SE = standard error, CI = confidence interval.

**Table 6:** Impact of the supplementation of the dam with *Saccharomyces cerevisiae* var. *boulardii* CNCM I-1079 on the probability of low birth weight puppies (*n* = 36 litters and 254 born alive puppies).

	Number of Q1 BWb		Multinomial regression	
	Control	Yeast	OR ± SE (95% CI)	*P*-value
**Treatment (control as reference)**	41	19	3.5 ± 1.77 (1.14–10.69)	0.028
**Age (class 1 as reference; ≤3 y)**				
Class 2 (>3 ≤ 4 years)	26	17	1.14 ± 1.95 (0.31–4.21)	0.844
Class 3 (>4 ≤ 6 years)	26	17	1.16 ± 1.97 (0.38–5.52)	0.580
**Litter size (class 1 as reference, <7 puppies born)**				
Class 2 (7–8 puppies born)	4	17	0.59 ± 2.24 (0.12–2.85)	0.508
Class 3 (> 8 puppies born)	4	39	0.21 ± 2.15 (0.05–0.94)	0.041

Q1 BWb = birthweight in the lowest 25%, OR = Odds Ratio, SE = standard error, CI = confidence interval.

Concerning puppy’s general health, no effect of yeast was obtained on APGAR score, rectal temperature or glycaemia (all *p* > 0.10) which averaged 8.2 ± 0.2, 36.1 ± 0.2°C, and 125.5 ± 5.4 mg/dL, respectively (data not shown). A total of 221 puppies were weaned resulting in a mortality rate from birth to DPP56 of 21.8%, with no difference between the two groups (Table 5).

### 3.3 Impact of supplementation on immunometabolic phenotype of puppies

#### 3.3.1 Postnatal growth of puppies

The supplementation of the bitches with the live yeast did not change the GR 0–21, while increasing the GR 21–56 (*p* < 0.05, Table 6). Interestingly, a significant effect of BWb on GR 0–21 was observed, with higher values in LBW categories (*p* < 0.01, Table 6 and Figure 3B). In addition, a tendency towards a greater GR 0–21 was observed in large-sized puppies compared with the other sizes (*p* < 0.1, Table 7).

**Table 7:** Impact of supplementation of the dam with *Saccharomyces cerevisiae* var. *boulardii* CNCM I-1079 on postnatal growth of puppies (*n* = 36 litters and 254 born alive puppies).

	LS means ± SE		*P*-value (fixed effects)				
	Control	Yeast	Treatment	Age	Breed size	Litter size	QBWb
GR 0–21, %	196 ± 12.2	178 ± 12.3	0.247	0.153	*0.054*	0.323	0.005
GR 21–56, %	190 ± 11.0	225 ± 11.2	0.022	0.334	0.393	0.393	0.742

GR x-y = growth rate between Day x and Day y, calculated as [(weight at Day y – weight at Day x) ÷ weight at Day x] × 100, LS means = adjusted least square means, SE = standard error, QBWb = quartile group of birthweight.

In addition, the variation coefficient of litters’ average body weight from DPP4 to DPP56 was significantly reduced in puppies from bitches of the Yeast group compared with those of the Control group (Figure 3C; asymptotic test for the equality of coefficients and modified signed-likelihood ratio test (SLRT) for equality of coefficients, *p* < 0.05). At the end of the trial (DPP56) variation coefficients of litters’ average BW were 33.2 and 14.2% for Control and Yeast group, respectively.

#### 3.3.2 Immune parameters

Before vaccination (DPP35), puppies from bitches of the Yeast group had higher serum concentrations of anti-inflammatory cytokine IL-10 and of TGFβ, while the 3 other pro-inflammatory cytokines were unaffected (Table 8). After vaccination (DPP56), serum concentrations of IL-8 tended to decrease while serum contents of IL-10 tended to increase in puppies from yeast-fed mothers, resulting in decreasing the IL-8: IL-10 ratio (Table 8). The evaluation of the specific immune response after vaccination measured by anti-rabies IgG titration revealed that whatever the experimental group all puppies were protected against the virus (Table 8).

**Table 8:** Impact of the supplementation of the dam with *Saccharomyces cerevisiae* var. *boulardii* CNCM I-1079 on cytokine levels (medians) of their puppies before and after vaccination against rabies (*n* = 57 puppies).

	DPP35, before vaccination			DPP56, after vaccination		
	Control (*n* = 31)	Yeast (*n* = 26)	*P*-value	Control (*n* = 31)	Yeast (*n* = 26)	*P*-value
**Serum cytokines, pg/mL**						
IL-8	4,401	3,828	0.975	2,768	2,128	*0.064*
IFNα	708.7	297.8	0.669	1,157	326.7	0.559
IFNγ	0	0	0.598	0	0	0.314
IL-10	247.4	531.3	0.043	168.9	407.6	*0.068*
TGFβ	1,300	1,640	0.001	1,421	1,327	0.754
IL-8:IL-10	14.7	7.77	0.241	16.9	4.22	0.032
**Specific IgG anti-rabies, UI/mL**						
IgG	0.13	0.13	0.200	3.46	5.29	0.936

## 4 Discussion

The gestation, peripartum and neonatal periods are challenging for bitches and their puppies. The aim of the present study was to evaluate whether supplementing the dams with live yeast from mid-gestation until separation of the puppies could support the animals during these periods. The effect of supplementation was investigated both in the dams and in their offspring.

Disruption of the maternal microbiota around birth could compromise normal bacterial colonization of the infant’s gastrointestinal tract, and, in turn, increase susceptibility to inflammation and reduce gut barrier function (52–54). Measuring the microbiota resilience is a good way to evaluate the impact of the *peri-partum* period, which is a physiological stress, on the health status of the bitches and their offspring (55). The diversity index has been described as a good indicator of gut resilience in humans (56, 57). Previous work in humans has shown that the diversity of bacterial species within an individual typically declines with gestation and this may contribute to microbiota unbalance (58), probably because of hormonal storm. In our study, in agreement with this observation, significant differences were identified in the Control group between G28 and G56 and then G56 and DPP1; the Shannon index being the lowest at G56. Thus, the supplementation with SB-1079 seemed to limit the variation in the microbiota diversity around parturition. Interestingly, same evolution of alpha-diversity is described in sows, for which microbiota richness is reported to be the lowest on day 1 before and day 3 after parturition, and such result has also been reported in sows (59, 60). Thus, adding SB-1079 before a physiological challenge like parturition can be an interesting strategy to improve microbiota resistance and/or recovery, both components of the resilience.

Overall, the microbiota of the bitches was characterized by ASVs typical of healthy adult dogs like Fusobacteriaceae, *Enterobacteriaceae*, *Clostridium hiranonis* (recently renamed as *Peptacetobacter hiranonis*), *Bacteroides*, *Phascolarctobacterium* (61), but also by other ASVs that are more typical of gestation and lactation, like *Lactobacillaceae*, *Porphyromonas* and *Corynebacterium*. Pregnancy and lactation periods in bitches are most probably accompanied by changes on the gut microbiota as noticed in other species. In humans and swine, these changes include increased abundance of *Proteobacteria*, *Actinobacteria*, and opportunistic pathogens and a decrease in species richness as well as in SCFA producers bacteria, all occurring as pregnancy progresses (60, 62). Interestingly, the beta-diversity was overall not different between the 2 groups but differed significantly one day *post-partum*.

At day 1 *post-partum* (Figures 2C,D; Supplementary Figure S2), feeding SB-1079 *pre-partum* promoted a higher relative abundance of bacteria known as beneficial and linked to SCFAs production, notably *C. hiranonis*, *F. prausnitzii*, *Dorea*, *Megamonas* and *Bacteroides plebeius*. *F. prausnitzii* is a pH and oxygen sensitive bacteria, which is a well described butyrate producer while *Fusobacterium* spp. are among the “canine heatlhy microbiota bacteria” and can produce butyrate from protein (61, 63). Increased abundance of *Megamonas* spp., an important propionate and acetate producer, has been found in healthy compared to diarrheic cats (64) and in dogs consuming inulin-rich diets (65). Interestingly in sows, a better persistence of relative abundance of fibrolytic bacteria, oxygen and pH sensitive strains, was reported in SB-fed sows (13, 59), suggesting that the live yeast can shape the microenvironment of the gut to favor growth of strict anaerobes. Besides the observed increase in beneficial bacteria, the LEfSe analysis at DPP1 also indicated a higher relative abundance of Enterobacteriaceae in the Yeast group compared to the Control group (Supplementary Figure S1). To deeply explore this difference, we conducted qPCR to evaluate the number of 16S DNA copies belonging to *E. coli* and found no difference between the two groups (data not reported here). All along the study, the microbiota of bitches fed the control diet was characterized by higher relative abundances of diverse bacteria (facultative anaerobes for most of them) known as opportunistic and potentially pathogenic like *Proteus* spp., *Mycoplasma* spp., *Tissierellaceae.ph2.ph2_un* and/or *Vagococcus* spp. compared with Yeast group. Another potential pathogen, *Tissierella* sp. was found in higher relative abundance in LBW puppies (66). Interestingly, Bendahmane et al. demonstrated that the supplementation of bitches with SB-1079 resulted in a decrease of opportunistic pathogenic bacteria relative abundances, especially in LBW puppies (67).

Secretory IgA (sIgA) are used as an intestinal biomarker because they play an important role in maintenance of gastrointestinal homeostasis by coating the bacteria, favoring a tolerant, non-inflammatory relationship with the host and the homeostatic control of the intestinal redox environment. Faecal sIgA concentration is well-correlated with the sIgA concentration in supernatants taken from duodenal explant cultures in dogs, thus measuring faecal sIgA provides a good indicator of mucosal immunoglobulin levels, reflecting what happens in the gut. In our study, SB-1079 supplementation of bitches resulted in the increase in faecal sIgA concentration (23.3 ± 3.17 vs. 14.5 ± 1.98 mg/g in average during all the study period), especially at DPP1. In agreement with these results, calves supplemented with SB have been shown to exhibit higher concentrations of ileal and colonic sIgA, along with a greater relative abundance of jejunal *F. prausnitzii* (68, 69). On the contrary, Meineri et al. (70) reported a decrease in faecal sIgA in adult dogs supplemented with SB. The sIgA production is still difficult to interpret, as it is intimately connected with stress hormones release, immune cells and microbiota. Nevertheless, decrease in faecal sIgA has been identified in case of stress challenge in rodent models, while higher concentrations have been described as providing a better protection against colonization of *Clostridium difficile* in infants (71). To go further and to fully understand the effect observed in our study, an evaluation of the concentration of butyrate produced as well as a characterization of the faecal IgA-binding bacteria would have been required. Taken all together, the higher faecal IgA concentrations and the positive microbiota changes could mean that SB may favor better colon homeostasis during the challenging period around whelping.

Bitches receiving SB-1079 in their food exhibited subtle modulation in their plasmatic metabolome just before parturition (Supplementary Figure S2). Interestingly, a part of the discriminant metabolites (glycerophospholipids, acylcarnitines, ketone bodies) between Yeast and Control groups seems to be associated to lipid and energy metabolisms. Two glycerophospholipids are found as discriminant between the 2 groups. Interestingly, Alassane-Kpembi et al. (72) reported that the supplementation of piglets with SB resulted in a modulation of blood and liver glycerophospholipid pathway. Acyl-carnitines derivatives are involved in fatty-acids β-oxidation and essential to meet the fetal energy needs and to fuel the placenta. When fatty acid oxidation is defective or diminished an increase in plasma acylcarnitine levels can be observed (73). In humans, several studies tried to find biomarkers of LBW or preeclampsia. The concentration of certain lysophosphatidylcholines in cord blood has been shown to positively correlate with BWb (74). Besides, the increase in serum levels of propionyl-carnitine, malonyl-carnitine, isovaleryl-carnitine, palmitoyl-carnitine and linoleoyl-carnitine were associated with gestational diabetes mellitus risk (75). Finally, ketone bodies are considered as markers of gestational diabetes during pregnancy in women (76). We could thus hypothesize that the supplementation with SB allow a better lipid/energy metabolic status, leading to better perinatal outcomes, i.e., the decreased odd of birth of LBW puppies or a higher gross energy in colostrum. However, those results are preliminary in canine species, and further research would be required to better measure the importance of those potential biomarkers of energy metabolism during gestation.

Another metabolite which raises interest during pregnancy is creatine, which has been found increased and discriminant in the Yeast group. Its homeostasis changes during pregnancy and alterations to maternal creatine homeostasis throughout gestation could impact the growth and well-being of the offspring. There is an increased requirement for maternal creatine due to the rapid growth and increased metabolic requirements of the fetus in the third trimester of pregnancy in humans. Interestingly, maternal urinary creatine excretion across pregnancy was positively correlated with birthweight centile and birth length, suggesting a relationship between maternal creatine status and fetal growth (77). In a retrospective case-controlled study (78), a 18% reduction in maternal serum creatine concentration during the third trimester of pregnancy was associated with a greater incidence of poor perinatal outcomes. Our study suggests for the first time and as in humans, a link between creatine metabolism during gestation and perinatal outcomes in canine species. Whether alterations in maternal circulating creatine concentrations are indicative of other poor perinatal outcomes is still to be ascertained. Thus, while links are beginning to emerge between maternal creatine homeostasis throughout pregnancy and infant outcomes, further studies are required to understand adaptations to maternal creatine homeostasis throughout gestation and their association with pregnancy outcomes. Supplementation with SB could offer a nutritional strategy for modulating creatine metabolism with the aim of improving perinatal outcomes.

Our study highlights that, in pregnant bitches like in women, there is an interdependence between the gut microbiota and the host. A better faecal microbiota resilience of the alpha-diversity occurs with a consecutive promotion of SCFA-producing bacteria in bitches fed with SB-1079. It would have been interesting to evaluate SCFA production in order to correlate it with the results obtained from microbiota and metabolomic analyses. However, data obtained constitute microbial indications suggesting a relationship between microbiota diversity, SCFAs and energetic metabolism. Coumarate, found as a discriminant metabolite, is a phenolic compound which has been found to be present in high quantity in the extracellular fraction of SB (79) produces through shikimate pathway, an active metabolic pathway in SB (80), and directly absorbed in the upper digestive tract. This compound possesses antioxidant activity, protects against generation of reactive oxygen species and has shown potential therapeutic benefit in experimental diabetes and hyperlipidemia (79, 81). Interestingly, supplementation of pregnant women with polyphenols have been reported to decrease the risk of intrauterine growth restriction (82). Another interesting metabolite that links microbiota and host-metabolism is the itaconate which has been recently discovered to be a mammalian antimicrobial metabolite. Itaconic acid has great potential to be an antimicrobial compound against viruses and antibiotic-resistant bacteria and exhibits immunomodulatory effects (83). Its production occurs after lipopolysaccharide (LPS) activation of macrophages (83) to compete against succinate in the tricarboxylic acid (TCA) cycle. A decrease in the plasmatic concentration of this compound could be related to a positive effect of SB in the gut, resulting in less LPS stimulation through toll-like receptor 2 (TLR2) (84). The discriminant metabolite indole-3-acetate is consistent with a positive effect on gut barrier, as indole and its derivatives may promote the upregulation of adhesion-related genes and enhance the intestinal barrier to prevent enteritis. Of note, the pathway of indolacetaldehyde production from tryptophan is an active pathway in SB (80). Also indole-3 acetate occurs from tryptophan breakdown by the gut bacterial activity [*Clostridium* (85) but also *Bifidobacterium* (86)].

Throughout gestation and then via mammary secretions, female provides her puppies with the energy they need to survive and develop. During the first days after birth, colostrum, providing energy and immunity, is indispensable for newborn puppies to deal with the challenging transition from intrauterine to extrauterine life (19, 87). Many studies in pigs support that live yeast supplementation during gestation and/or lactation improves the quality of colostrum (16, 88–91). In the current study, the SB-1079 supplementation had no effect on the immune composition of the colostrum. Results of other studies using live yeast are controversial. No change in the immune composition was described in dairy cows (92) or in sows (13) but another study in sows demonstrated an increase of the IgG concentration in sow colostrum (93). Additional studies on the impact of SB supplementation on the immune composition of the colostrum in the canine species are required due to its great variation among bitches. Indeed, the IgG and IgA concentrations may vary greatly depending on time elapsed since the onset of lactation and sampling, and depending on the number of puppies born within a litter and their suckling behaviour (33, 94). However, the supplementation of bitches with SB-1079 increased colostrum gross energy (Table 4). It could be beneficial considering the high energy requirements of puppies just after birth, their limited energy reserves and their immature thermoregulation system (95). Interestingly, in bitches, plasma N-acetylneuraminate (also known as sialic acid) was identified as a discriminant metabolite, suggesting a modulation of amino-sugar metabolism (96, 97). Sialic acid has been described as increasing in gestating women, being largely involved in the milk oligosaccharides synthesis, and crossing the placenta to participate to *in utero* body growth and brain development (98). Dunière et al. (91) reported that a supplementation of gestating ewes with a live yeast resulted in significantly higher levels of colostral N-glycolylneuraminic acid. In our study, the decrease in number of LBW puppies as well as the trend to have more sugars in the colostrum are consistent with a modulation of the amino-sugar metabolism. In addition, we previously observed that 2-day old puppies from bitches supplemented with SB had a faecal microbiota richer in Lactibacillaceae which are well-described to use milk oligosaccharides (67). Finally, at day 7 *post-partum*, SB-fed bitches had a higher concentration of milk proteins and a trend of higher energy content. It was described that in the canine species, proteins provide a significant portion of the calories in colostrum and milk (99). The modulation of the plasma metabolome just before parturition could relate to the increase in energy and protein content in the mammary gland secretions, although further research is required for a better understanding of the mechanisms involved. Alternatively, it is possible that supplementation with SB improved overall nutrient digestibility in the bitches (92).

The concept of immunometabolism has emerged recently whereby the repolarizing of inflammatory immune cells toward anti-inflammatory profiles by manipulating cellular metabolism represents a new potential approach to control inflammation. It is of great interest to study it during the early life. Interestingly, main mechanisms described to sustain the DOHaD hypothesis (6) are related to epigenetics, for which mitochondrial metabolites and cofactors play a critical role and for which microbiota has been demonstrated to be a histone methylation and acetylation driver (100). B vitamins group (including nicotinic acids) and SCFAs are for example sources of acetyl-CoA or nicotinamide adenine dinucleotide (NAD) which affect histone acetylation while butyrate induced histone acetylation (100). Thus, supplementing bitches with live yeast SB-1079 could affect the immunometabolic profile of their offsprings, via different mechanisms. In the current study, this profile was evaluated through the body weight at birth, the growth rate, the specific immune response as well as plasma pro- and anti-inflammatory cytokines before and after vaccination against rabies. Further studies are warranted to deeply explore the persistence of metabolic changes observed in puppies, through for example modification of the epigenome (101).

One parameter of litter composition, the repartition of puppies among BWb quartiles, was significantly influenced by the SB-1079 supplementation. Reducing the number of LBW puppies is of great interest for breeders considering their lower energy reserves, lower motility, higher risks of hypoxia, hypothermia and thus, their higher risk of neonatal mortality compared to normal BWb (20, 21, 102). Apart from this major short-term consequence, LBW was also suggested to increase odds to become obese in later life in the canine species (8). Further studies are needed to understand how supplementation could influence intra-uterine growth, especially as this result has not been found in other studies on pigs. One hypothesis could be that SB supplementation improved overall nutrient digestibility by modulation of dam microbiota and improvement of metabolism (103). The potential antioxidant effect of SB through its metabolites, as well as the effect on energy metabolism suggested in our study could also allow an increase of nutrients dedicated to fetus growth or a better placental function or efficiency (103). Future studies should therefore include an assessment of antioxidant status as well as an evaluation of nutrient digestibility in bitches to support the hypotheses suggested by the results of the current study.

Beside the effect on BWb quartiles, an impact in the growth rate pattern was detected, with notably an improved growth during the second part of the suckling period. As in humans, monitoring the growth of puppies is recommended to identify abnormal patterns. This should be used as a proxy to evaluate short- and long-term health (104). To our knowledge, those results are the first ones reporting such an effect of live yeast supplementation to bitches on the growth pattern of puppies. A rapid growth at the beginning of life is not desired as it may favor obesity in later life (105–107). In dogs, the link between early postnatal growth and later overweight as well as the sensitivity window in which rapid growth should be avoided remain to be explored. Interestingly, our study confirmed that puppies belonging to the LBW quartile (Q1) exhibited a quicker, also called “catch-up,” growth compared to the other puppies, already described in rodents or humans (108, 109). Therefore, further studies are required to evaluate the effect of the improved growth detected in the yeast group during the second half of the suckling period on long-term health. Besides the impact on the pattern, yeast supplementation allowed a better homogeneity of postnatal growth between puppies and thus avoided extreme trajectories. Having a homogenous growth pattern in the kennel should also facilitate litter’s management for breeders. In our study, puppies were not directly fed with SB, leading to speculate an indirect effect of SB. In swine, SB given to the sows has also been demonstrated to have a beneficial effect on the postnatal growth of piglets. This could be explained by the improved immune status, assessed by the serum IgG concentration, observed in piglets born to supplemented dams (16–18).

In parallel to the growth patterns, inflammatory and immunological status of the puppies was assessed before and after vaccination against rabies virus. This antigen was chosen to make sure that all the dogs, including bitches, were naïve. The aim was to avoid interference with maternal antibodies, but also to ensure that the reaction to the vaccine was not biased by a reaction to the pathogen linked to spontaneous infection. Just before vaccination, the plasma concentrations in IL-10 and in TGF-β were significantly increased when bitches received SB-1079. After vaccination, while the specific immune response was efficient in all puppies, blood IL-8 tended to decrease; blood IL-10 to increase, leading to a significant lower IL-8: IL-10 ratio. The supplementation of the bitches with SB-1079 attenuated the inflammatory response after a vaccination while maintaining appropriate levels of specific IgG. In humans fed SB and suffering from diarrhea-dominant irritable bowel syndrome blood IL-10 increased; while blood IL-8 and TNF-α decreased (110) whereas in sheep, a direct supplementation induced higher seroconversion after vaccination (73). The IL-8 plays a critical role in acute inflammation and its concentration increases in blood of infants suffering from necrotizing enterocolitis (111). More generally, circulating IL-8 is proposed as a promising biomarker of gut permeability in humans (112). Besides, IL-10 and TGF-β are associated with immune tolerance and promotion of Treg cells and are thus of paramount importance during early life.

## 5 Conclusion

Our study highlighted an effect of SB-1079 on bitches’ outcomes and in turn on maternal programming of puppies, confirming that like in other species, maternal nutrition can impact the immunometabolic phenotype of the offsprings. Indeed, in dams, it resulted in modulating gut homeostasis and microbiota while modifying cell metabolism. The inter-talk between microbiota and host seems to be an interesting area to be further investigated, in this physiological condition, but also in others like obesity, insulin resistance or in elderly populations. Feeding bitches with SB-1079 is a promising approach to optimize the health and nutrition of the vulnerable mother–infant dyad, but continued work in nutritional immunology is required to further enhance our understanding of the power of nutrition and diet to improve early-life health of companion animals.

Interestingly, a lot of similarities between humans and dogs have been highlighted, the canine species appearing as a good model to understand the DOHaD in humans and to test the effect of some nutritional strategies. More research on gut microbiota and blood or urinary metabolism of mothers and puppies is required to better understand the maternal programming effects allowed by the supplementation of live yeast to the bitches. To compare with what happens in humans, it could be of great interest to monitor the impact of the SB supplementation of the bitches on the long-term health of puppies, and notably on the obesity and diabetes risk assessment which is a major issue in pets as well as in human population.

## Data availability statement

The datasets presented in this study can be found in online repositories. The names of the repository/repositories and accession number(s) can be found at: https://www.ncbi.nlm.nih.gov/bioproject/PRJNA1063569/.

## Ethics statement

The animal study was approved by Comité d’Éthique en Expérimentation Animale, Science et Santé Animale n°115; reference number: SSA_2020-004, Toulouse, France. The study was conducted in accordance with the local legislation and institutional requirements.

## Author contributions

QG: Conceptualization, Data curation, Formal analysis, Investigation, Methodology, Project administration, Resources, Validation, Visualization, Writing – original draft, Writing – review & editing. AM: Formal analysis, Writing – original draft, Writing – review & editing. SC: Conceptualization, Funding acquisition, Investigation, Supervision, Writing – review & editing. FS: Formal analysis, Investigation, Visualization, Writing – review & editing. J-CM: Formal analysis, Investigation, Visualization, Writing – review & editing. LS: Formal analysis, Investigation, Visualization, Writing – review & editing. MC: Funding acquisition, Supervision, Writing – review & editing. MR-V: Formal analysis, Investigation, Writing – review & editing. NR: Resources, Writing – review & editing. LM: Formal analysis, Resources, Writing – review & editing. PD: Resources, Writing – review & editing. EM: Resources, Writing – review & editing. AR: Data curation, Formal analysis, Visualization, Writing – review & editing. HM: Conceptualization, Funding acquisition, Investigation, Project administration, Supervision, Writing – review & editing. EA: Conceptualization, Formal analysis, Funding acquisition, Project administration, Supervision, Visualization, Writing – original draft, Writing – review & editing.
