# Supplementary material for: The supplementation of female dogs with live yeast Saccharomyces cerevisiae var. boulardii CNCM I-1079 acts as gut stabilizer at whelping and modulates immunometabolic phenotype of the puppies
Source: Front Nutr. 2024 Apr 12;11:1366256. doi: 10.3389/fnut.2024.1366256 (PMC11048480; doi:10.3389/fnut.2024.1366256)
Supplement: Supplementary file 1 [file Table_1.DOCX]

**Supplementary files**

**Table S1. Veterinary protocol of the breeding facility.**

| **Dog** | **Period** | **Veterinary treatment** |
| --- | --- | --- |
| Adults | 1 month before ovulation | **Vaccines**  *-*Parvovirus (attenuated virus, Vanguard, Zoetis, France);  -Canine distemper, Rubarth hepatitis (attenuated type 2 adenovirus);  -Parvoviruses (attenuated type 2b parvovirus);  -Parainfluenza, and leptospirosis (attenuated *L. interrogans* serovar icterohaemorrhagiae, serovar Bratislava and serovar canicola, and *L. kirschneri* serovar grippotyphosa; Biocan Novel DHPPi+L4 vaccine, Bioveta, Czech Republic) |
|  | Entering the maternity building, around 8 days before parturition | **Antiworm**  Pulverization of 1ml/L water of phoxime, (Sebacil 50% solution 500 mg/mL, Elanco, Germany) |
| Puppies | Between DPP9 and DPP33 of age | **Antiworm**  Two doses, per os, at one week of interval. 50 mg/kg fenbendazole (Panacur, MSD Animal Health, UK) and 1 mg/kg of diclazuril (Vecoxan, MSD Animal Health, France) |
|  | DPP21 of age | **Vaccines**  *Bordetella bronchiseptica* and parainfluenza (Nobivac KC vaccine, MSD animal health) |
|  | DPP28 of age | **Vaccines**  Parvovirus (Vanguard, Zoetis, France; 3 injections at weekly intervals) |

In addition, when necessary, some puppies received medicinal treatments consisting of antibiotics (amoxicillin and clavulanic acid - Synulox (Zoetis, Malakoff, France); metronidazole - Flagyl (Sanofi, Gentilly, France) or Eradia (Virbac, Carros, France)), accompanied or not with anti-inflammatory drugs (meloxicam - Metacam (Boerhinger Ingelheim Animal Health France, France)) and/or of digestive aid products (Lafaure SAS, bentonite and smectite).

**Table S2. Thresholds of birth weight used to create four quartile of birth weight for each breed, and number of birth weight used to calculate thresholds.**

| **Breed** | **Q1** | **Q2** | **Q3** | **Q4** | **Number of past birth weights used to calculate thresholds** |
| --- | --- | --- | --- | --- | --- |
| **Australian Shepherd** | < 325 g | 364-378 g | 378-426 g | > 426 g | 663 |
| **Golden Retriever** | < 389 g | 389-438 g | 438-498g | > 498 | 249 |
| **Labrador Retriever** | < 336 g | 336-382 g | 382-425 g | > 425 g | 1094 |
| **White Swiss Shepherd** | < 364 g | 364-410 g | 410-455 g | > 455 g | 713 |

**Table S3. Type of ELISA, sensitivity, detection rank and detection wavelength for each cytokine assayed**.

| Cytokine | ELISA | Sensitivity | Rank | wavelenght |
| --- | --- | --- | --- | --- |
| IL-8 | sandwich | 0.146 ng/mL | 0.312 ng/mL-20 ng/mL | 450 nm |
| IL-10 | sandwich | 0.39 pg/mL | 1.56 pg/mL-100 pg/mL | 450 nm |
| IFN-α | sandwich | 3.12 pg/mL | 12.5 pg/mL-800 pg/mL | 450 nm |
| IFN-γ | sandwich | 0.78 pg/mL | 3.12 pg/mL-200 pg/mL | 450 nm |
| TNF-α | sandwich | 0.039 ng/mL | 0.156 ng/mL-10 ng/mL | 450 nm |
| TGF-β | sandwich | 0.747 ng/ml | 0.781 ng/ml - 50 ng/ml | 450 nm |

**Table S4. Parameters used for the data processing in the four analysis modes used in the study.**

| XCMS parameters | C18 positive | C18 negative | HILIC positive | HILIC negative |
| --- | --- | --- | --- | --- |
| peak detection method | Cent Wave | Cent Wave | Cent Wave | Cent Wave |
| peak width | 3, 40 | 2, 30 | 3, 60 | 4, 80 |
| noise | 50000 | 12000 | 50000 | 40000 |
| prefilter | 4, 150000 | 3, 36000 | 4, 150000 | 4, 120000 |
| ppm | 5 | 5 | 5 | 5 |
| peak alignement | Obiwarp | Obiwarp | Obiwarp | Obiwarp |
| peak grouping method | density | density | density | density |

**Table S5. Breeds and size of the dogs included in the experiment and their repartition between the treatment groups.**

| **Breed** | **Size** | **Control** | **Additive** |
| --- | --- | --- | --- |
| Australian shepherd | Medium | 4 | 4 |
| Bernese Mountain dog | Large | 4 | 2 |
| Boxer | Large | 0 | 1 |
| Dalmatian | Medium | 0 | 1 |
| German shepherd | Large | 1 | 0 |
| Golden Retriever | Large | 2 | 6 |
| Labrador | Large | 4 | 2 |
| Patou | Large | 2 | 0 |
| Swiss White Shepherd | Large | 1 | 2 |

**Table S6. Characteristics of the cohort before the supplementation with the yeast, at 28 days of gestation.**

| **Group** | **N** | **Mean** | **SD** | **Median** | **Min** | **Max** | **P-value^1^** |
| --- | --- | --- | --- | --- | --- | --- | --- |
| **Age** | | | | | | | |
| Control | 18 | 3.55 | 1.18 | 3.35 | 1.20 | 5.90 | 0.275 |
| Yeast | 18 | 3.11 | 1.19 | 3.05 | 1.10 | 5.90 |  |
| **Parity** | | | | | | | |
| Control | 18 | 2.89 | 3.00 | 1.75 | 0 | 5.00 | 0.441 |
| Yeast | 18 | 2.44 | 2.00 | 2.01 | 0 | 6.00 |  |
| **Body Condition Score (BCS)** | | | | | | | |
| Control | 18 | 5.22 | 1.11 | 5.00 | 4.00 | 8.00 | 0.588 |
| Yeast | 18 | 4.89 | 1.18 | 5.00 | 3.00 | 7.00 |  |
| **Faecal score** | | | | | | | |
| Control | 18 | 3.17 | 0.62 | 3.15 | 2.20 | 4.20 | 0.349 |
| Yeast | 18 | 3.37 | 0.61 | 3.30 | 2.50 | 4.30 |  |

^1^ Nonparametric Wilcoxon-Mann-Whitney rank test between yeast and control groups.
