# Supplementary figures and images for: The supplementation of female dogs with live yeast Saccharomyces cerevisiae var. boulardii CNCM I-1079 acts as gut stabilizer at whelping and modulates immunometabolic phenotype of the puppies
Source: Front Nutr. 2024 Apr 12;11:1366256. doi: 10.3389/fnut.2024.1366256 (PMC11048480; doi:10.3389/fnut.2024.1366256)

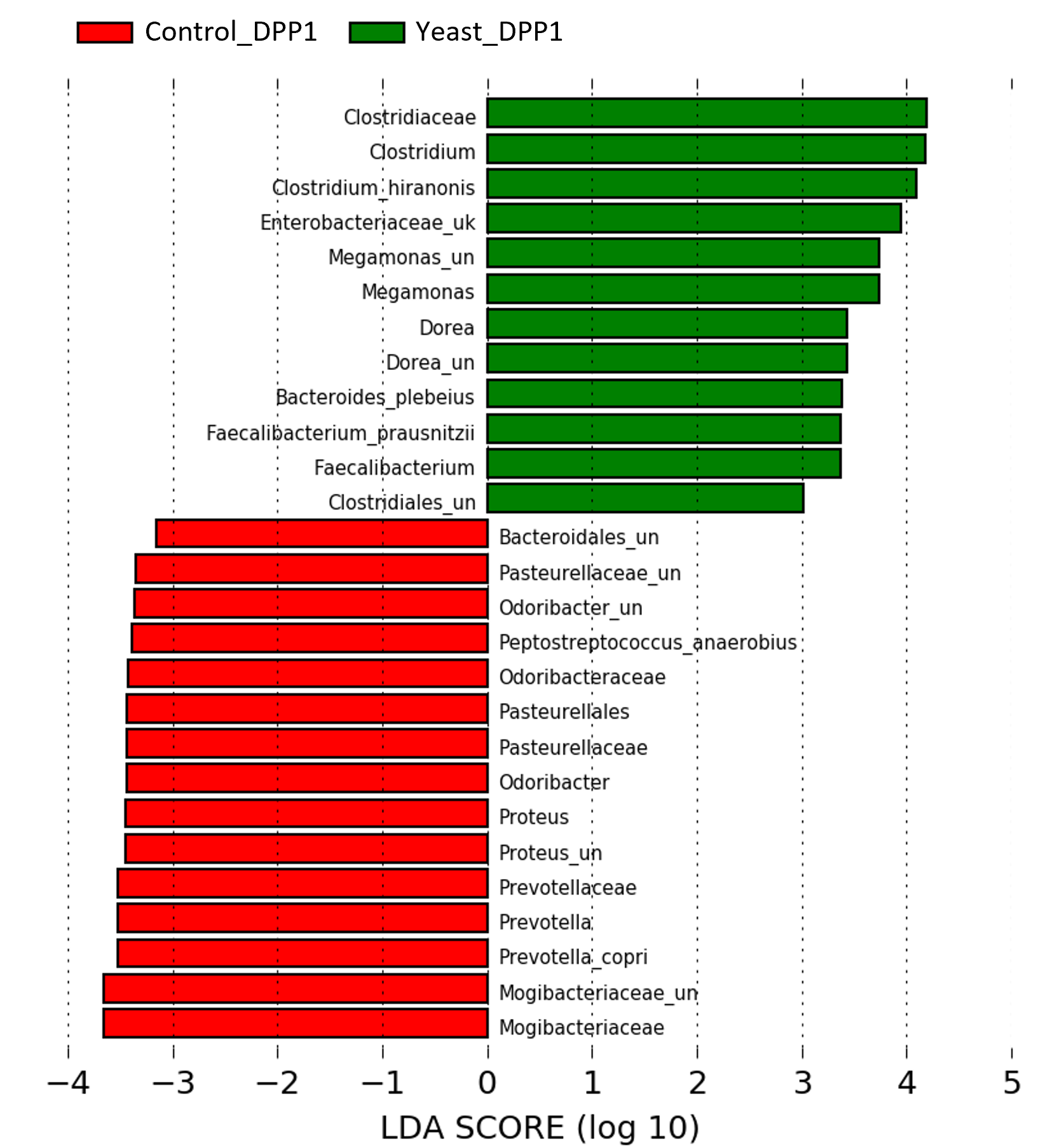

Supplement: Supplementary file 2 [file Image_1.TIF]

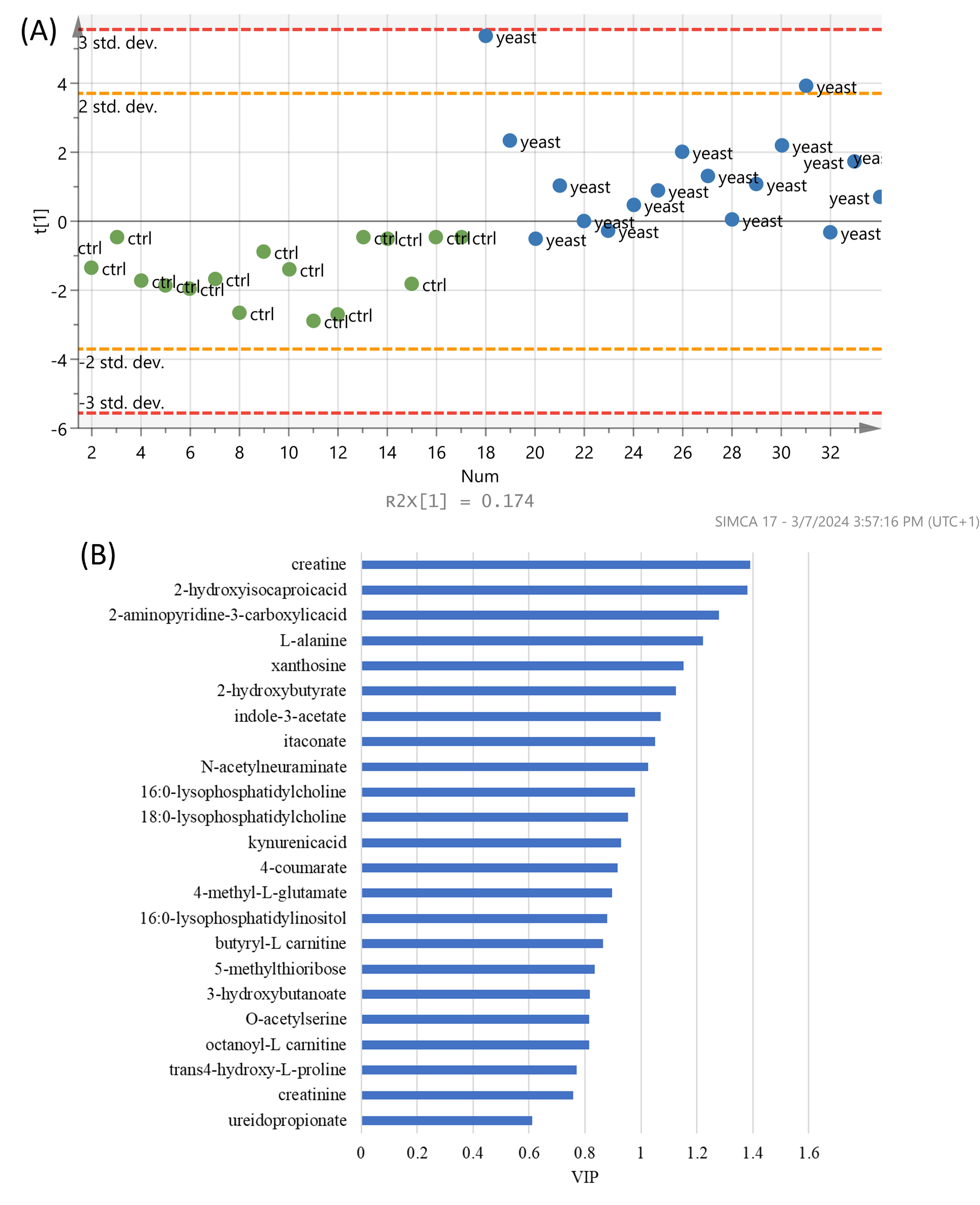

Supplement: Supplementary file 3 [file Image_2.TIF]
